# Polycomb-like Proteins in Gene Regulation and Cancer

**DOI:** 10.3390/genes14040938

**Published:** 2023-04-18

**Authors:** Sabrina Fischer, Robert Liefke

**Affiliations:** 1 Institute of Molecular Biology and Tumor Research (IMT), Philipps University of Marburg, 35043 Marburg, Germany; 2 Department of Hematology, Oncology and Immunology, University Hospital Giessen and Marburg, 35043 Marburg, Germany

**Keywords:** Polycomb-like, PHF1, MTF2, PHF19, cancer, Polycomb repressive complex 2, chromatin, transcription, epigenetics, leukemia

## Abstract

Polycomb-like proteins (PCLs) are a crucial group of proteins associated with the Polycomb repressive complex 2 (PRC2) and are responsible for setting up the PRC2.1 subcomplex. In the vertebrate system, three homologous PCLs exist: PHF1 (PCL1), MTF2 (PCL2), and PHF19 (PCL3). Although the PCLs share a similar domain composition, they differ significantly in their primary sequence. PCLs play a critical role in targeting PRC2.1 to its genomic targets and regulating the functionality of PRC2. However, they also have PRC2-independent functions. In addition to their physiological roles, their dysregulation has been associated with various human cancers. In this review, we summarize the current understanding of the molecular mechanisms of the PCLs and how alterations in their functionality contribute to cancer development. We particularly highlight the nonoverlapping and partially opposing roles of the three PCLs in human cancer. Our review provides important insights into the biological significance of the PCLs and their potential as therapeutic targets for cancer treatment.

## 1. Introduction

Failures in chromatin and gene regulatory mechanisms can be the source of various human diseases [1]. Polycomb repressive complex 2 (PRC2) is a key component of the chromatin regulatory machinery of the cell [2,3]. The primary function of PRC2 is to add methyl groups to lysine 27 of histone H3 (H3K27) to establish H3K27 trimethylation (H3K27me3), which induces gene repression. PRC2 is particularly relevant for maintaining the correct gene expression pattern during development [2] and is often associated with cell differentiation [3].

Given its central role in gene regulation, dysregulation of PRC2 is commonly observed in various cancer types [2,4,5]. In solid tumors, such as prostate cancer, bladder cancer, and melanoma, high PRC2 activity is typically associated with aggressive and advanced disease progression [5]. Thereby, overexpression of PRC2 leads to the repression of tumor suppressor genes, which can contribute to the development and progression of these cancers [5]. In contrast, in some cancer types, such as in T-ALL (T-cell acute lymphoblastic leukemia) [6,7] and breast cancer [8], PRC2 activity is reduced, leading to enhanced expression of oncogenes.

In some cancer types, loss- or gain-of-function mutations of the core components of PRC2 are observed. Many mutations affect the histone methyltransferase EZH2, such as in hematologic malignancies [7,9,10,11], which globally influence the chromatin landscape [7,12]. Furthermore, the target of PRC2, the amino acid K27 of histone 3, can be altered. In pediatric glioblastoma, a mutation to methionine (H3K27M) is commonly observed [13,14] and prevents the efficient spread of H3K27me3, a requirement for tumorigenesis in this cancer type [15].

The activity of PRC2 is mainly determined by the expression of its core components, which facilitate the enzymatic deposition of the H3K27me3 mark. However, not only the PRC2 core determines the activity of PRC2 but also the associated factors influence the composition, recruitment, and functionality of PRC2. Here, we describe and discuss the role of the Polycomb-like proteins (PCLs) in regulating PRC2 and how their dysregulation contributes to the development and progression of human cancer.

## 2. Polycomb-like Proteins Are Key Components of the PRC2.1 Subcomplex

Research in recent years has demonstrated that mammalian PRC2 represents not a single complex but a variety of alternative complexes that possess a common core complex but differ in the accessory factors [16]. The number of PRC2 components has also considerably increased during evolution, leading to a greater variety of possible compositions of the mammalian PRC2 compared to nonvertebrate PRC2 [17].

The catalytic core of PRC2 consists of four components: The histone methyltransferase EZH2 is responsible for the deposition of the majority of the H3K27me3 histone mark. EZH1, the homolog of EZH2, is structurally similar to EZH2 but is less essential for H3K27me3 deposition [18]. Instead, EZH1 may play a role in chromatin compaction by establishing dimeric PRC2 [19]. EZH2 alone cannot perform efficient H3K27me3 methylation but requires additional factors [20]. Another critical component of PRC2 is SUZ12, which acts as a scaffold that brings all necessary proteins together [16,21]. It allows the binding of EZH2, EED, and one of the two highly homologous proteins, RBBP4 and RBBP7 [21]. RBBP4/7 are structural components of the PRC2 complex, but are not required for its enzymatic activity [20]. EED also has a structural function [22]; additionally, it also recognizes the substrate of PRC2, H3K27me3, which is vital for the activity of PRC2 on chromatin [23]. Only the combination of EZH2, SUZ12, and EED establishes the necessary structural conformation that allows efficient methylation of histone proteins [16,22].

**Figure 1:**
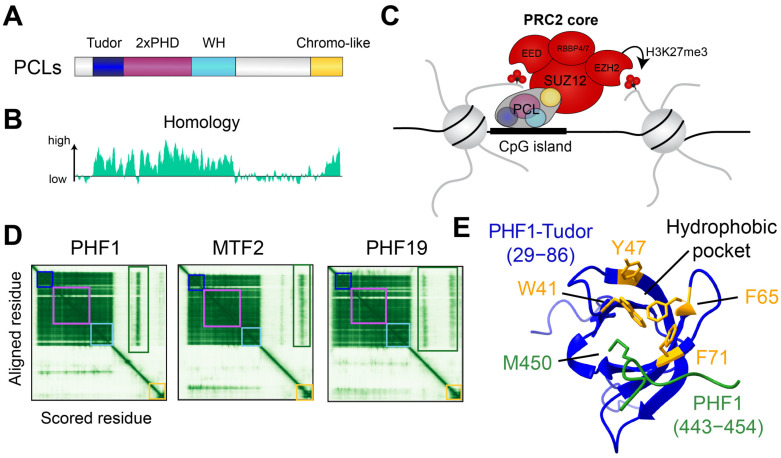
(**A**) General domain structure of the three PCLs. (**B**) Amino acid homology between the three human PCLs. (**C**) Model of the functional role of the PCLs for recruiting PRC2. PCL interacts via the winged helix domain with unmethylated CpG islands and recruits PRC2 by interaction of the C-terminal chromo-like domain with the PRC2 core. The histone methyltransferase EZH2 facilitates H3K27me3 methylation. EED interacts with H3K27me3 and contributes to PRC2 recruitment, as well. (**D**) Predicted aligned error of human PHF1, MTF2, and PHF19 obtained from the AlphaFold database [31]. Green rectangles indicate predicted associations of the unstructured regions with the chromatin-binding internal domains. (**E**) AlphaFold2 predicted the association of the amino acids 443–454 with the Tudor domain of PHF1. Amino acids forming the hydrophobic pocket are marked yellow. Structure obtained from the AlphaFold database of human PHF1 (O43189, version 2022-11-01) [31].

The PRC2 core complex is associated with additional proteins, establishing alternative subcomplexes [16,17]. The two major PRC2 subcomplexes are named PRC2.1 and PRC2.2 and are defined based on the proteins that are associated with the PRC2 core. The PRC2.2 subcomplex possesses JARID2 and AEBP2 [16,17] and will not be further addressed here. The defining characteristic of PRC2.1 is the presence of one of the PCL proteins, which is mutually exclusive to the binding of JARID2 and AEBP2 [17,24]. In the mammalian system, three PCL proteins exist: PHF1, MTF2, or PHF19 (alternative names are PCL1, PCL2, and PCL3, respectively). These three PCL proteins have evolved from the same ancestor protein, called Polycomb-like (Pcl) [25], and consequently have a similar domain composition [17]. They contain five different conserved domains (Figure 1A,B). In the central part of the protein, the PCLs possess a large globular region that consists of a Tudor domain, two PHD fingers, and a winged helix (WH) domain. This large domain is the main chromatin-binding module of the PCL proteins [26]. In addition to this sizeable globular domain in the center, the PCLs possess a conserved domain at the very C-terminus, which holds distant similarity with chromodomains [27]. This domain has been demonstrated to be essential for interaction with the PRC2 core [21,27].

Although the PCL proteins are the key features defining the PRC2.1 subcomplex, they are not the only associated proteins found explicitly with PRC2.1. In addition to the PCLs, PRC2.1 can also be associated with either EPOP [28,29] or PALI1 [30] or their homologous proteins, SKIDA1 and PALI2, respectively [17,24]. The specific role of these proteins regarding PRC2 and how they may work together with the PCLs in the context of PRC2.1 are not fully understood and will not be addressed further in this review.

## 3. Polycomb-like Proteins in PRC2 Chromatin Recruitment and Mammalian Gene Regulation

PCLs are crucial in targeting PRC2 to chromatin and, thus, important for gene repression (Figure 1C). Deleting all three PCL homologs substantially reduces the chromatin association of PRC2 [32,33], suggesting that PRC2 chromatin binding strongly depends on the PCLs.

The PRC2 recruitment function of the PCLs particularly relies on the DNA-binding ability of their winged helix (WH) domains (Figure 1C). The WH domain recognizes explicitly unmethylated CpG motifs via a positively charged region that reaches deeply into the major groove of the DNA [26]. The presence of methylated cytosine leads to a steric clash, preventing DNA binding [26]. This binding property is consistent with the common presence of PRC2 at unmethylated CpG islands in the genome [34]. Notably, the three PCLs proteins differ in their ability to interact with DNA. While MTF2 and PHF1 can strongly bind to DNA, the DNA binding function of PHF19 is more subtle [26]. Consistently, in mouse embryonic stem cells, the recruitment of PRC2 to CpG islands is particularly dependent on MTF2 [32], the most strongly expressed PCL protein in these cells. Consequently, mutation of the DNA-binding ability of MTF2 substantially reduces the PRC2 recruitment function of MTF2 in these cells [26]. It has also been proposed that the MTF2 DNA-binding function is linked to a specific helical confirmation of the DNA [35]. Thus, these findings suggest that the DNA-binding ability constitutes a major feature of the PCLs by which they are involved in recruiting PRC2 to its genomic targets.

The role of the other domains is less well understood. The Tudor domains of all three mammalian PCLs form a hydrophobic cage and have a high affinity for trimethylated lysine 36 of histone H3 (H3K36me3) [36,37,38,39]. PHF1 and PHF19 can also recognize the K27 trimethylation of the testis-specific histone variant H3t [40,41]. However, the binding strength of the Tudor domain to this modification is substantially lower than that for H3K36me3, suggesting that H3K36me3 is the preferred target of the Tudor domain. Nonetheless, the relevance of the H3K36me3 binding function of the Tudor domain remains controversial, given that H3K36me3 is a transcription elongation mark found in the gene body, and PRC2 typically does not colocalize with H3K36me3 in the genome. Thus, it is unclear whether this histone-binding ability is important for the chromatin recruitment of PRC2 or whether it may have another function related to H3K36me3. Given that H3K36me3 has been found to inhibit the enzymatic activity of PRC2 [42,43], the H3K36me3-binding function of the Tudor domain could potentially also play a role in inferring with the activity of PRC2 at active genes. In addition, the PHD finger 1 of PHF1 has been proposed to interact with symmetrically methylated arginine at position 3 of histone 4 (H4R3me2s) [44]. Whether the PHD fingers of PHF19 and MTF2 have merely a structural function or whether they are also directly involved in chromatin binding has not yet been determined.

To recruit PRC2 to its target genes, the PCL must tightly interact with the PRC2 core complex. This function is facilitated via the very C-terminal chromo-like domain of the PCLs, (Figure 1A), which is necessary and sufficient for interaction with PRC2 [21,27]. The C-terminal domain is sequence-wise and similar between MTF2 and PHF19, consists of one helix and two β sheets, and shares some similarities with the chromodomains of HP1 (heterochromatin-protein 1) [27]. The C-terminal domain of PHF1 lacks the α helix [27], which may suggest some alternative structural functionality. For PHF19 and MTF2, it has been shown that the domain directly associates with the N-terminal part of SUZ12 [45] and that this association enhances the establishment of a dimeric state of PRC2, which increases its affinity for CpG islands and chromatin [45].

Notably, the region between the chromatin-binding internal domain, comprising the Tudor, PHD fingers, and the winged helix domain and the C-terminal PRC2-interacting domain consists of a long unstructured stretch, which does not appear to establish any globular domain (Figure 1D). This region differs substantially among the three PCLs in their amino acid sequences and shows almost no homology (Figure 1B). Furthermore, AlphaFold2 [31,46] predicts that this unstructured region may contact the internal domain (Figure 1D), which could potentially be important for modulating the chromatin-binding function. For example, in the case of PHF1, but not for MTF2 and PHF19, this predicted association is close to the hydrophobic pocket of the Tudor domain (Figure 1E). This raises the possibility that the histone reader function of the Tudor domain could be influenced by such an association.

In summary, the PCLs link the PRC2.1 subcomplex to chromatin by interacting with the PRC2 core via the C-terminal chromo-like domain [27,45] and by interacting with DNA [26] and histones [27,37,38,39] via their internal domain (Figure 1C). However, the functions of the domains vary in detail, suggesting that PCLs may have nonredundant roles in regulating PRC2.

## 4. Polycomb-like Proteins in Human Cancer

**Figure 2:**
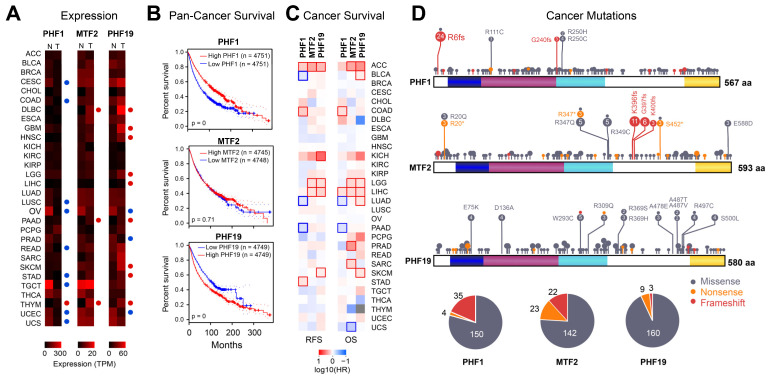
(**A**) Comparison of gene expression of the PCL proteins in normal (N) and tumor (T) tissue. Red and blue circles indicate significantly increased or decreased gene expression in tumor versus normal samples, respectively. (**B**) Kaplan–Meier plot of pancancer patient survival (overall survival) dependent on the expression of PHF1, MTF2, and PHF19. The data were split based on median expression. (**C**) Survival plots showing the hazard ratio (HR) for the three PCLs for individual cancer types. RFS = relapse free survival and OS = overall survival. Cancers with rectangles indicate a significant difference in survival between low and high expression. Data from TCGA [48] and visualized by GePIA [49]. (**D**) Mutations of the three PCL proteins, based on the COSMIC database [47]. Visualized using ProteinPaint [50]. * = mutation to a stop codon, fs = frameshift mutation. The pie charts show the relative distribution of missense, nonsense, and frameshift mutations for each PCL protein.

An investigation of the role of the PCLs in cancer using publicly available databases suggests that the three PCLs have nonoverlapping roles in cancer (Figure 2). First, the expression changes in cancer versus normal tissue considerably differ between the proteins (Figure 2A). PHF1 is predominantly downregulated in cancer, while PHF19 is largely upregulated [28], implicating a potentially opposing role of these two proteins. Indeed, when looking at all cancer types, high PHF1 expression is associated with a better prognosis, while high PHF19 expression is typically linked to a worse outcome (Figure 2B). MTF2 expression is not strongly affected in most cancer types, and its expression does not correlate with patient survival (Figure 2A,B). Notably, however, the pattern of the gene expression changes, and the correlation with patient survival is variable and dependent on the individual cancer type and on the investigated PCL protein (Figure 2A,C). For example, a high PHF1 expression is linked to a worse prognosis in colon adenocarcinoma (COAD) but a better prognosis in pancreatic cancer. In addition, only a few cancer types, such as ovarian cancer (OV) and thymoma (THYM), show a similar pattern for all three PCLs (Figure 2A,C). In most other cancer types, the role of the PCLs is highly individual. In addition to changes in gene expression, all three PCL proteins are also commonly mutated in cancer (Figure 2D) [47]. Currently, the specific consequences of these mutations are largely unknown. Interestingly, for PHF1 and MTF2 frameshift or nonsense mutations, which lead to truncated proteins, are often observed. Especially the recurrent frameshift at the amino acid R6 of PHF1 (Figure 2D) suggests that abolishing the function of PHF1 is advantageous for cancer progression, consistent with commonly observed reduced gene expression of PHF1 (Figure 2A).

Thus, although these data globally support a tumor-suppressive role of PHF1, an oncogenic role of PHF19, and a relatively neutral role of MTF2, the three PCL proteins appear to have individual impacts dependent on the cancer type. In the following, we will address the known roles of PHF1, MTF2, and PHF19 in cancer and the possible mechanisms involved.

### 4.1. Tumor-Suppressive and Oncogenic Roles of PHF1

Public data suggest that PHF1 is typically downregulated in many cancer types (Figure 2A), and its low expression is often associated with a poorer prognosis (Figure 2B). In addition, in some cancer types, such as carcinomas [47], frameshift mutations are commonly observed (Figure 2D). This indicates a more tumor-suppressive role of PHF1. Indeed, several publications support this idea, and it has been shown that the tumor-suppressive function of PHF1 is linked to its ability to regulate p53-dependent pathways in cancer [51]. P53 is one of the most common tumor-suppressor genes in cancer, and inactivating mutations are often crucial for cancer development [52]. The interaction of PHF1 stabilizes p53 by preventing MDM2-dependent ubiquitination and proteasomal degradation [51]. Thus, the downregulation of PHF1 in cancer cells could be involved in reducing the protein level of p53 and thereby impairing its tumor-suppressor activity. Additional work suggests that the link between PHF1 and p53 depends on two unique serines in the N-terminal PHD finger of PHF1. These serines are not present in MTF2 or PHF19 [53], explaining why the interaction with p53 is restricted to PHF1. The PHF1–p53 axis is essential for inducing cellular quiescence and to prevent uncontrolled cell growth [53]. One mechanism that regulates PHF1 expression levels in cancer involves the RNA-binding protein FTO, which stabilizes the *PHF1* mRNA [54]. PHF1 has also been shown to be involved in DNA repair mechanisms via interaction with the Ku70/Ku80 heterodimer [38,55]. Therefore, PHF1′s tumor-suppressive role may also be linked to its ability to suppress DNA damage and thereby prevent harmful chromosome rearrangement.

Although PHF1 appears to function mostly as a tumor suppressor, in the context of breast cancer cells, PHF1 has also been described as promoting cellular proliferation [44]. Here, PHF1 was found to interact not only with the PRC2 complex but also with the PRMT5/WDR77/CRL4B complex, which is involved in histone arginine methylation and functions as an H2AK119ub reader. Like the association with p53, this interaction appears to be specific for PHF1.

Thus, PHF1 can interact with additional factors not related to PRC2, such as p53 [51,52,53,56], PRMT5 [44], and Ku70/Ku80 [55], leading either to a tumor-suppressive or an oncogenic role, depending on the cellular context.

### 4.2. PHF1 in Gene Fusions

**Figure 3:**
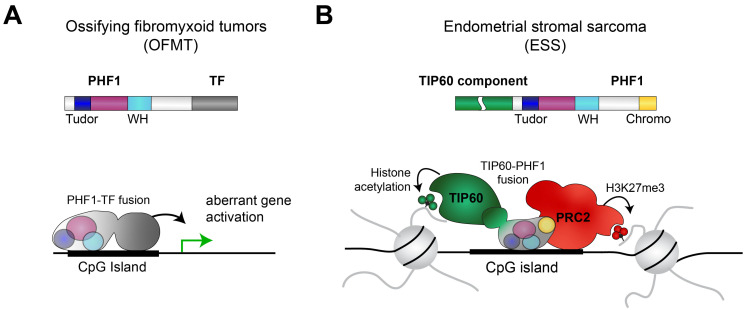
(**A**) Common PHF1 rearrangements in OFMT consist of the N-terminal part of PHF1 and transcription factors (TF), such as FOXR1, FOXR2, and TFE3. Consequently, the resulting fusion protein may be aberrantly recruited to the wrong locations in the genome, leading to dysregulated gene expression. (**B**) In ESS, full-length PHF1 is recurrently fused to components of the TIP60 complex, such as TEAF1, JAZF1, EPC1, and EPC2 (see also Table 1). The resulting protein can interact with both the TIP60 and the PRC2 complexes [62], leading to aberrant chromatin states in cancer cells.

In addition to the role of PHF1 itself, PHF1 is also commonly involved in gene rearrangements in some cancer types. To date, eleven in-frame gene fusions have been described (Table 1, Figure 3). Most of these rearrangements were identified in ossifying fibromyxoid tumor (OFMT) or endometrial stromal sarcoma (ESS), both relatively rare cancer diseases.

OFMT is a soft-tissue tumor characterized by bone-like tissue formation within the tumor [57]. In approximately 50% percent of the OFMT cases, the PHF1 gene is rearranged [58], often with a transcription factor gene, such as *FOXR1* [59], *FOXR2* [59], or *TFE3* [60]. In these cases, the N-terminal chromatin-binding region of PHF1 is translocated to the transcription factor in frame, resulting in a transcription factor that possesses an additional chromatin-binding function. These fusion proteins lack the C-terminal PRC2-interacting domain of PHF1, suggesting that these fusion proteins act in a PRC2-independent manner. One could speculate that the fusion protein can be recruited to places normally targeted by PHF1, such as CpG islands [26]. The displaced transcription factor recruitment to these locations may lead to the dysregulation of target genes relevant to the development of OFMT. This idea is supported by the observed gene expression changes upon ectopic expression of a PHF1-TFE3 fusion protein [61].

ESS is a relatively rare malignant tumor of the uterus, accounting for approximately 0.2% of all uterine malignancies [63]. In ESS, most known translocation partners of PHF1 are linked to the NuA6/TIP60 histone acetyltransferase complex [62]. This includes EPC1, EPC2, EP400, MEAF6, MBTD1, ING3, BRD8, and JAZF1. In most cases, the entire PHF1 is fused to one of the TIP60 components. The EPC1-PHF1 fusion protein has been demonstrated to assemble a supercomplex consisting of both the TIP60 complex and PRC2 [62]. Expression of the EPC1-PHF1 fusion proteins leads to dysregulation of gene expression, particularly of the PRC2 target genes, which become upregulated [62]. This finding can be explained by the observation that the EPC1-PHF1 fusion protein is recruited to PRC2 target genes and enhances the histone acetylation level, likely due to the activity of TIP60 [62]. Similar supercomplexes are likely also formed by the other PHF1 fusion proteins in ESS, as well as by other rearrangements, such as the JAZF1-SUZ12 translocation. These observations suggest that bringing together TIP60 and PRC2 via a gene fusion is a common event in ESS [62].

**Table 1:** Recurrent PHF1 gene rearrangements in cancer.

Translocation	Cancer Type	Reference
PHF1-FOXR1	ossifying fibromyxoid tumors	[59]
PHF1-FOXR2	ossifying fibromyxoid tumors	[59]
PHF1-TFE3	ossifying fibromyxoid tumors, malignant chondroid syringoma	[60,64,65]
MBTD1-PHF1	endometrial stromal sarcoma	[66]
JAZF1-PHF1	endometrial stromal sarcoma cardiac ossifying sarcoma	[67,68,69]
EPC1-PHF1	endometrial stromal sarcoma ossifying fibromyxoid tumors	[62,67,70]
EPC2-PHF1	endometrial stromal sarcoma	[71]
EP400-PHF1	ossifying fibromyxoid tumors	[72,73]
MEAF6-PHF1	ossifying fibromyxoid tumors endometrial stromal sarcoma	[70,72,74,75,76]
ING3-PHF1	endometrial stromal sarcoma	[77]
BRD8-PHF1	endometrial stromal sarcoma	[78,79]

### 4.3. A Predominantly Tumor-Suppressive Role of MTF2 in Cancer

To date, the physiological function of MTF2 has been predominantly linked to its role in stem cell maintenance and differentiation [26,80,81] and during hematopoiesis [82]. The cancer-related role of MTF2 appears rather versatile and can both promote and inhibit tumor growth.

In acute myeloid leukemia, a reduced level of MTF2 has been associated with increased chemotherapy resistance [83], thus resulting in a worse prognosis. In AML cells, it has been demonstrated that MTF2, together with PRC2, inhibits the expression of MDM2, a key regulator of the tumor suppressor p53. Consequently, it has been proposed that either overexpression of MTF2 or inhibition of MDM2 sensitizes the leukemic cells to chemotherapeutics [83], offering a potential new strategy for treating AML patients. A similar link between low MTF2 expression and increased resistance to chemotherapeutics has been shown in basal-like breast cancer cells [84]. Here, reduced activity of PRC2 led to the derepression of Nfat1c, which is important for epithelial–mesenchymal transition (EMT) and resistance to cytotoxic treatments. Another study suggests that MTF2 may influence the MDM2-p53 axis in breast cancer cells, similar to AML cells [56]. Low expression of MTF2 triggered by miR-218-5p has also been linked to increased epithelial–mesenchymal transition, cell migration, and invasion in retinoblastoma [85]. Thus, most studies propose a more tumor-suppressive role of MTF2.

However, some studies also suggest an oncogenic role of MTF2. MTF2 was identified as a candidate gene in myeloma using Myc-transgenic mice in an unbiased genetic forward screen [86]. Indeed, a high expression of MTF2 is linked to a poorer patient prognosis in this cancer type, and a depletion of MTF2 leads to reduced proliferation of cells in vitro and in xenograft experiments [86], supporting a putative oncogenic role of MTF2 in this context. In hepatocellular carcinoma (HCC), the high expression of MTF2 has been linked to the activation of EMT-related pathways, whereby Snail was identified as an important downstream effector of MTF2 [87], further supporting that MTF2 can act as an oncogene in some cancer types.

### 4.4. The Oncogenic Role of PHF19

In contrast to PHF1 and MTF2, PHF19 has primarily been described as an oncogene in multiple cancer types [88]. Of note, in humans, but not in mice, PHF19 has a shorter isoform, which contains the Tudor domain and the first PHD finger but lacks the second PHD finger, the winged helix domain, and the PRC2-interacting chromo-like domain [89]. Consistently, this isoform does not interact with PRC2 [90], suggesting that this isoform has a distinct functionality from full-length PHF19. Currently, many studies regarding PHF19 in cancer do not fully distinguish between these two isoforms, making the conclusion about the role of PHF19 in cancer less defined.

Initially, PHF19 was identified as a gene similar to Drosophila PCL, which shows elevated expression levels in cancer [89]. This upregulation is particularly evident for the short isoform but also measurable for the long isoform. The first functional study in cancer about PHF19 was performed in melanoma cells, where depletion of PHF19 leads to reduced proliferation but enhances invasiveness [91], suggesting a nontrivial role of PHF19. A similar role in regulating invasiveness was subsequently found in mammary tumors [92].

To date, the most compelling role of PHF19 in cancer has been found in multiple myomas (MM) [93]. In this cancer type, PHF19 is required for setting up PRC2-dependent broad H3K27me3 domains, which is important for the optimal silencing of PRC2 target genes [94]. The expression of PHF19 in multiple myoma cells is negatively regulated by miRNA-15a [95]. The relevance of PHF19 in this cancer type is also supported by computational approaches [89,96], functional assays [94,97], and patient data [98].

In the context of prostate cancer, a study demonstrated that the depletion of the full-length PHF19 unexpectedly leads to increased chromatin binding of PRC2 [90]. An enhanced chromatin binding of MTF2 in the absence of PHF19 explains this finding [90]. Consequently, the deletion of PHF19 leads to the substantial deregulation of multiple pathways, including those involved in cell growth, metastasis, and blood vessel formation. Interestingly, the absence of PHF19 changes the properties of the cells to a less proliferative but, at the same time to a more aggressive phenotype, similar to the effect in melanomas [91]. Another study suggests that the short isoform of PHF19 is also important for the proliferation and migration of prostate cancer cells, suggesting a sophisticated role of PHF19 in this cancer type [99].

A study in hepatocellular carcinoma (HCC) suggests that a high expression of PHF19 promotes cell migration and invasiveness but also proliferation. The expression of PHF19 has here been linked to the expression of the tumor-suppressive miRNA hsa-miR-195-5p [100]. A similar link between an miRNA and a PHF19 has been proposed in ovarian carcinoma, where PHF19 expression is regulated by miR-211 [101].

In colorectal cancer, the *PHF19* gene appears to possess an oncogenic super-enhancer [102], a key feature in gene regulation [103,104]. The super-enhancer at *PHF19* likely drives a high expression of PHF19 in this cancer type, required for efficient tumor growth [102]. An oncogenic role of PHF19 has also been demonstrated in Ewing Sarcomas, where the PHF19 gene is downstream of the EWS/ETS fusion proteins that typically drive the oncogenic program in this cancer type [105]. Further studies suggest a role of PHF19 in other cancer types, such as ovarian and gastric cancer, for which PHF19 has mostly been described to function as an oncogene (Table 2).

PHF19 is relevant not only in the cancer cells themselves but also in cells involved in the fight against cancer. Namely, a high expression of PHF19, triggered by miR-155, in CD8+ T cells supported an antitumor response [106]. Notably, this function depends on the chromatin-binding ability of the Tudor domain of PHF19 [27,36,106], supporting the biological relevance of the chromatin-binding ability of the Tudor domain.

In summary, most studies on the role of PCL proteins in cancer refer to the oncogenic role of PHF19 in a variety of cancers and PHF1 gene translocations in endometrial stromal sarcoma and ossifying fibromyxoid tumors. Given that human PCL proteins have just emerged as interesting proteins in cancer, many more insights can be expected in the future.

**Table 2:** Roles of the PCLs in cancer.

Cancer Type	Expression Regulated by	Tumor Role	Reference
PHF1			
breast cancer	n.d.	mixed	[44,51]
lung adenocarcinoma	FTO	tumor-suppressor	[54]
MTF2			
acute myeloid leukemia	n.d.	tumor-suppressor	[83]
basal-like breast cancer	n.d.	tumor-suppressor	[84]
breast cancer	n.d.	tumor-suppressor	[56]
glioma	n.d.	oncogene	[107]
hepatocellular carcinoma	n.d.	oncogene	[87]
myeloma	n.d.	oncogene	[86]
retinoblastoma	n.d.	tumor suppressor	[85]
PHF19			
chronic myeloid leukemia	n.d.	oncogene	[108]
colorectal cancer	n.d.	oncogene	[102,109]
Ewing sarcoma	EWS/ETS fusion TFs	oncogene	[105]
gastric cancer	n.d.	oncogene	[110]
glioblastoma	n.d.	oncogene	[111,112]
glioma	miR-124a	oncogene	[113]
hepatocellular carcinoma	miR-195-5p	oncogene	[100,114]
mammary tumor	n.d.	mixed	[92]
melanoma	n.d.	mixed	[91]
multiple myeloma	miR-15a	oncogene	[93,94,95,96,97,98]
ovarian carcinoma	miR-211	oncogene	[101,115]
prostate cancer	n.d.	mixed	[90,99]

n.d. means not determined.

## 5. Conclusions

Polycomb-like proteins are key components of the Polycomb repressive complex 2 and are the defining factors for the PRC2.1 subcomplex. They are essential for targeting PRC2 to chromatin via the histone-binding Tudor [27,36,37] and the DNA-binding winged helix domains [26]. Although the three PCL proteins have a similar domain composition, their biological roles are not identical and differ in physiological and nonphysiological settings.

The difference in functionality can partly be explained by their distinct ability to interact with other proteins beyond PRC2. In particular, PHF1 has been described to interact with PRC2-unrelated proteins, including p53 [53] and Ku70/Ku80 [55]. This unique interaction of PHF1 allows it to participate in PRC2-independent biological pathways. PHF19 has been described to interact with the histone lysine demethylase RIOX1 (NO66) [36], while for MTF2, no PRC2-unrelated interacting partners have been described so far, suggesting that their functions are likely mostly linked to their chromatin function. Another reason for their distinct functionality is that the DNA-binding winged helix and the histone-binding Tudor domain differ in their binding strength [26,39]. Consequently, the ratio of the three PCL proteins in a specific cell may be critical to determine which PCL preferentially binds to the PRC2 core, which in turn influences the extent and where PRC2 is recruited. Beyond these known differences, the variation in the primary amino acid sequence of the PCLs likely contributes to other currently unknown differences in their functionality.

Thus, it is unsurprising that each of the three PCL proteins has a distinct role in cancer. While PHF1 has a predominantly tumor-suppressive role linked to its ability to interact with p53, PHF19 acts mainly as an oncogene (Table 2). Furthermore, PHF1 is recurrently translocated in two rare cancer types: ossifying fibromyxoid tumors and endometrial stromal sarcoma (Table 1). The highly specific occurrence of these translocations further emphasizes the context dependency of the PCLs in cancer.

Taken together, PCLs play complex and diverse roles in cancer, and their precise functions can vary depending on the specific type of cancer and the stage of the disease. Further research is needed to fully understand the mechanisms by which the PCLs contribute to cancer development and progression and to identify strategies to influence their functions in cancer.

## Data Availability

The data used in this article were obtained from the UniProt (www.uniprot.org (accessed on 19 March 2023)), GePIA (http://gepia.cancer-pku.cn/ (accessed on 19 March 2023)) and AlphaFold databases (https://alphafold.ebi.ac.uk/ (accessed on 19 March 2023)).
